# Intraperitoneal adoptive transfer of mesenchymal stem cells enhances recovery from acid aspiration acute lung injury in mice

**DOI:** 10.1186/s40635-017-0126-5

**Published:** 2017-03-06

**Authors:** Tommaso Mauri, Vanessa Zambelli, Claudia Cappuzzello, Giacomo Bellani, Erica Dander, Marina Sironi, Vittoria Castiglioni, Andrea Doni, Alberto Mantovani, Andrea Biondi, Cecilia Garlanda, Giovanna D’amico, Antonio Pesenti

**Affiliations:** 1 Department of Anesthesia, Critical Care and Emergency, Fondazione IRCCS Ca’ Granda Ospedale Maggiore Policlinico, Via F. Sforza 35, 20122 Milan, Italy; 2 School of Medicine and Surgery, University of Milan-Bicocca, Monza, Italy; 3 Research Center ‘M. Tettamanti’, Fondazione MBBM/San Gerardo Hospital, Monza, Italy; 4 Humanitas Clinical and Research Center, Rozzano, MI Italy; 5 Mouse and Animal Pathology Lab, Fondazione Filarete, Milan, Italy; 6 Department of Pathophysiology and Transplantation, University of Milan, Milan, Italy

**Keywords:** Acute respiratory distress syndrome, Acute lung injury, Stem cells, Pentraxin 3, Acid aspiration syndrome

## Abstract

**Background:**

Mesenchymal stem cells (MSCs) might act as fine-tuners of inflammation during acute lung injury. We assessed the effects of adoptive transfer of MSCs in acid aspiration acute lung injury and explored the role of long pentraxin PTX3.

**Methods:**

We conducted a prospective experimental interventional study on wild-type (WT) and PTX3-deficient (PTX3^−/−^) mice. Acute lung injury was induced in WT and PTX3^−/−^ mice by instillation of hydrochloric acid into the right bronchus. One hour later, animals received intraperitoneal sterile phosphate-buffered saline (PBS), WT-MSCs (1 × 10^6^) or PTX3^−/−^-MSCs (1 × 10^6^). Twenty-four hours after injury, we measured the effects of treatments on arterial blood gases, wet/dry lung weight (W/D), CT scan analysis of lung collapse, neutrophils, TNFα and CXCL1 in bronchoalveolar lavage, and plasma PTX3. d-dimer was assayed in 1 week and OH-proline in 2 weeks to track the fibrotic evolution.

**Results:**

In 24 h, in comparison to PBS, WT-MSCs improved oxygenation and reduced W/D and alveolar collapse. These effects were associated with decreased concentrations of alveolar neutrophils and cytokines. WT-MSCs increased d-dimer concentration and decreased OH-proline levels, too.

Treatment with PTX3^−/−^-MSCs ameliorated oxygenation, W/D, and alveolar TNFα, though to a lesser extent than WT-MSCs. PTX3^−/−^-MSCs did not improve lung collapse, neutrophil count, CXCL1, d-dimer, and OH-proline concentrations. The protective effects of WT-MSCs were dampened by lack of endogenous PTX3, too.

**Conclusions:**

In acid aspiration acute lung injury, MSCs improve pulmonary function and limit fibrosis by fine-tuning inflammation. The role of PTX3 in determining MSCs’ effects might merit further scrutiny.

**Electronic supplementary material:**

The online version of this article (doi:10.1186/s40635-017-0126-5) contains supplementary material, which is available to authorized users.

## Background

The incidence of the acute respiratory distress syndrome (ARDS) is elevated, and mortality in recent studies still reaches 50% for the most severe form [1–5]. Moreover, many ARDS survivors develop long-term lung fibrosis, reduced respiratory function, and poor quality of life [1]. At onset, ARDS is characterized by severe hypoxemia and lung edema, caused by dysregulated inflammation [1, 2]. Overstimulation of leukocytes, cytokine storm, and altered tissue repair are key contributors to ARDS severity, mortality, and long-term morbidity [3]. However, we still lack effective pharmacological therapies that fine-tune these mechanisms [4].

Mesenchymal stem cells (MSCs) are multi-potent cells derived from adult tissues [6]. MSCs secrete multiple molecules, including anti-inflammatory cytokines, growth factors, and anti-microbial peptides, and appear as fine-tuners of host inflammation [6]. Previous studies showed that MSCs administration in animal models of acute lung injury increased the ability of the host to eliminate the agent, regulate neutrophil recruitment, and reverse altered lung permeability, without additional injury [7–10]. In addition, intraperitoneal (i.p.) route for the administration of MSCs was recently described [11]. To our knowledge, the effects of i.p. MSCs have never been assessed in experimental acid aspiration acute lung injury [12]; moreover, the effects of MSCs on the fibrotic long-term evolution of acute lung injury [13] have not been described, and key molecular determinants of MSCs’ effects are not fully understood.

In the present study, we tested in a mouse model of acid aspiration acute lung injury the effects of i.p. MSCs on the early acute inflammatory reaction and on the long-term fibrotic evolution [5, 12]. Moreover, we explored the role of pentraxin 3 (PTX3) in mediating MSCs’ effects. PTX3 is an acute-phase inflammatory mediator produced by different cell types [3, 14] that exerts protective effects in experimental acute lung injury, closely resembling those of MSCs [15]. Previous studies indicated that MSCs produce, store, and secrete PTX3 when activated [16, 17]. The research group of Dr. G. D’Amico generated PTX3-deficient MSCs (PTX3^−/−^-MSCs) [18], which showed a significant defect in promoting tissue repair in a mice model of wound healing compared to wild-type MSCs (WT-MSCs) [18]. In analogy, we investigated whether PTX3 deficiency in the MSCs and/or at the endogenous level might impact the ability of MSCs to promote short- and long-term recovery from acid aspiration acute lung injury.

The hypothesis of this study was that early treatment with MSCs in a murine model of acid-induced lung injury might exert short- and long-term beneficial effects by modulation of the inflammatory response and that lack of PTX3 in MSCs might reduce their efficacy.

## Methods

### Ethics and permissions

Procedures involving animals and their care were conducted in conformity with the institutional guidelines complying with national and international laws and policies. The experimental protocol was submitted to the Italian Ministry of Health and approved by the Animal Care Unit of the University of Milan-Bicocca, Monza, Italy.

### Isolation of MSCs

MSCs were isolated from female C57Bl/6 WT mice and from PTX3^−/−^ mice by previously described procedures [18]. Cryopreserved aliquots of MSCs were thawed 5–7 days before the experiments, seeded at 1000–2000 cells/cm^2^, and cultured at 37 °C in a 5%-CO_2_ atmosphere. On early morning, MSCs were dethatched by trypsin and used fresh for all the experiments performed that same day. Fresh MSCs at passages 5 to 7 were used for the present study. Recent studies showed that PTX3^−/−^-MSCs were similar to WT-MSCs in their ability to grow spontaneously, undergo mesengenic differentiation, and express common MSCs’ markers [18]. As already published, PTX3^−/−^-MSCs drastically decreased the mitogen-induced proliferation of lymphocyte in a dose-dependent manner similarly to WT-MSCs [19]. Moreover, PTX3^−/−^-MSCs did not store or release PTX3 while they tended to produce higher levels of tumor necrosis factor-stimulated gene 6 (TSG-6) [19] compared to WT-MSCs (Additional file 1: Figure S1).

### Experimental protocol

Acid aspiration acute lung injury was induced in WT- and PTX3^−/−^-mice as previously described [12]. Briefly, after intubation, 1.5 ml/kg of 0.1 M hydrochloric acid was instilled into the right lung, and after 10 min, the animals were extubated and placed in an oxygenated chamber. One hour later (to reproduce possible real life clinical timing), the mice received i.p. injection of sterile phosphate-buffered saline (PBS) or 1 × 10^6^ WT-MSCs or 1 × 10^6^ PTX3^−/−^-MSCs (all in equal volume of 200 μl).

### Experimental design

**Fig. 1:**
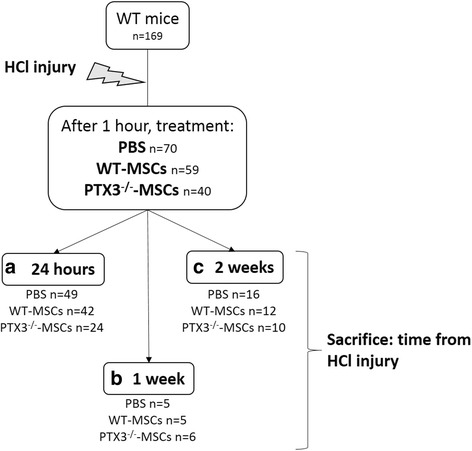
Experimental design. Experimental groups and number of animals (i.e., WT mice) studied at different time-points (injury, treatment, and sacrifice)

Figure 1 shows the experimental design of the study in WT mice. The following measures were performed in all WT mice:

- Twenty-four hours after HCl instillation, the mice were sacrificed and the following analysis were performed (detailed methods are described in Additional files):
  Arterial blood gas analysis for gas exchange
  Wet-to-dry ratio as index of edema
  Micro-CT scan to measure change over time in non-aerated lung tissue expressed as percentage of the whole lung tissue, with more negative values representing larger decrease of alveolar collapse;
  Histopathology examination performed according to previous study [12] evaluating alveolar serofibrinous exudate and alveolar hemorrhage
  Bronchoalveolar lavage for differential cell count, total protein content (with bicinchoninic acid method) and keratinocyte chemoattractant (CXCL1, previously named KC), and tumor necrosis factor-α (TNF-α) were assayed by ELISA
  Blood withdrawal for PTX3 levels measurement in plasma (ELISA assay)
- In 1 week from lung injury d-dimer (marker of fibrinolysis) [20] and matrix metalloproteinase 13 (MMP13), an enzyme that participates in collagen degradation [21], were detected by ELISA and by western blot in lungs lysate, respectively.
- Two weeks after acid-induced lung injury, the fibrotic evolution was evaluated [22]. In particular, we performed as follows:

In PTX3^−/−^-mice, instead, we measured only oxygenation and wet-to-dry lung weight ratio in 24 h and OH-Pro content in 2 weeks. Blinded researchers performed each analysis.

### Statistical analysis

Data are expressed as mean ± standard deviation if normally distributed and as median (interquartile range) when non-normally distributed. One-way analysis of variance (ANOVA) or Kruskal–Wallis and Dunnett’s or Dunn’s post hoc tests vs. PBS group were used to assess differences between treatment effects in WT mice, as appropriate. Differences in physiologic variables measured in the right vs. left lung were assessed by *t* test or Mann–Whitney *U* test, as appropriate. *p* < 0.05 was considered statistically significant.

Detailed methods can be found in the Additional file 1 of this article.

## Results

### Mesenchymal stem cells enhance short- and long-term recovery from experimental acid aspiration acute lung injury

**Fig. 2:**
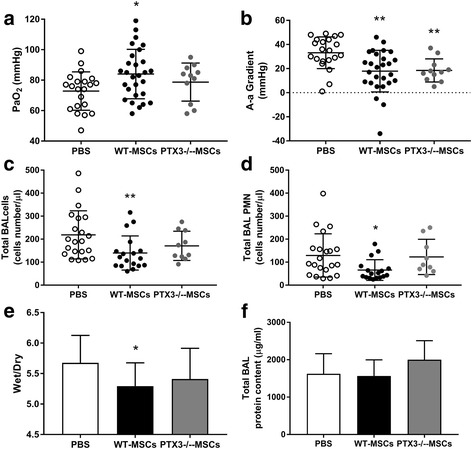
Early effects of mesenchymal stem cells (MSCs) on oxygenation, lung edema, and alveolar inflammatory cells in acid aspiration acute lung injury. Wild-type MSCs ameliorated arterial oxygen tension (**a**) and alveolar–arterial oxygen gradient (**b**) in experimental model of acid aspiration lung injury in 24 h, as well as PTX3-deficient MSCs, albeit to a lesser extent (Kruskal–Wallis *p* < 0.05 [A] and *p* = 0.001 [B]; Dunn’s post hoc *p < 0.05 and **p < 0.01 vs. PBS. PBS *n* = 21; WT-MSCs *n* = 28; PTX3^−/−^-MSCs *n* = 11). Total cell count (**c**) and total neutrophil (PMN) count (**d**) in the broncho-alveolar lavage (BAL) were decreased by WT-MSCs but not by PTX3^−/−^-MSCs in experimental groups in 24 h (Kruskal–Wallis *p* < 0.05 [C] and *p* = 0.01 [D]; Dunn’s post hoc *p < 0.05 and **p < 0.01 vs. PBS. PBS *n* = 21; WT-MSCs *n* = 17; PTX3^−/−^-MSCs *n* = 9). WT-MSCs significantly reduced lung edema (**c**), as measured by wet-to-dry lung weight ratio (wet/dry) in the experimental groups in 24 h (ANOVA *p* < 0.05 [**e**]; *Dunnett’s post hoc *p* < 0.05 vs. PBS. PBS *n* = 21; WT-MSCs *n* = 18; PTX3^−/−^-MSCs *n* = 10). No difference was seen in BAL total protein concentrations (**f**) (PBS *n* = 22; WT-MSCs *n* = 20; PTX3^−/−^-MSCs *n* = 10). (PBS = acid aspiration acute lung injury + intraperitoneal (i.p.) PBS treatment in 1 h; WT-MSCs = acid aspiration acute lung injury + i.p. wild-type MSCs treatment in 1 h; PTX3^−/−^-MSCs = acid aspiration acute lung injury + i.p. PTX3-deficient MSCs treatment in 1 h)

**Fig. 3:**
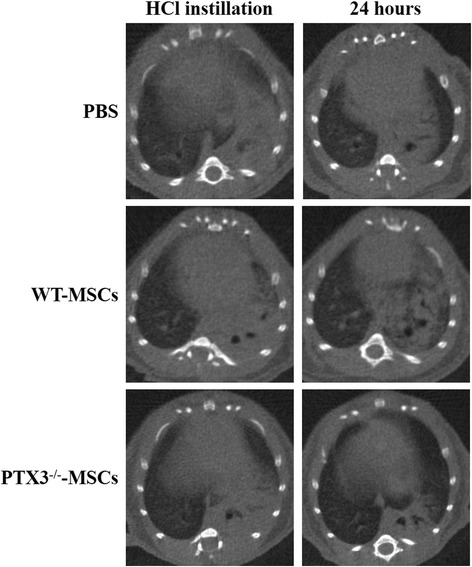
Short-term effects of MSCs: CT scan images. Chest CT images from representative study animals treated by PBS, WT-MSCs, or PTX3^−/−^-MSCs in 1 h after HCl instillation and 24 h showing reduced radiologic signs of lung edema and collapse, especially in the right lung

In 24 h, i.p. administration of WT-MSCs 1 h after induction of acid aspiration acute lung injury significantly improved arterial oxygenation and decreased the alveolar–arterial oxygen gradient in WT-mice in comparison to PBS (*p* < 0.05 and *p* = 0.001, respectively) (Fig. 2a and b), without modification of PaCO_2_ and even in presence of slightly worse pH values (Additional file 1: Table S1). Early improvement in oxygenation yielded by WT-MSCs was likely obtained by reduction of lung edema: in fact, the lungs’ wet-to-dry ratio in 24 h was decreased by WT-MSCs in comparison to PBS (*P* < 0.05) (Fig. 2e). Similarly, micro-CT scan analysis showed that the extent of lung collapse significantly decreased between 1 and 24 h in WT mice treated by WT-MSCs (*p* = 0.01), likely indicating decreased superimposed weight from reduced lung edema (Table 1 and Fig. 3), but not in those treated by PBS. Histology performed in 24 h showed decreased disruption of lung structures in mice treated by WT-MSCs in comparison to PBS (Table 1), even though this difference did not reach statistical significance. BAL total protein concentrations were left unchanged by WT-MSCs treatment (Fig. 2f). Mice treated by WT-MSCs, indeed, showed significant reduction of total cell count in BAL fluid in 24 h and substantial dampening of neutrophil recruitment into the alveoli (*p* < 0.05 for both; Fig. 2c, d) in comparison to PBS. Accordingly, levels of proinflammatory cytokines (i.e., CXCL1 and TNF-α) in BAL fluid were significantly reduced by WT-MSCs (*p* < 0.05 and *p* < 0.01, respectively), but not by PBS (Table 1). Interestingly, circulating PTX3 was reduced in WT-mice treated by WT-MSCs (albeit non-significantly) and not in WT-mice treated by PTX3-deficient MSCs (Table 1).

**Table 1:** Micro-computerized tomography (micro-CT scan) analysis, histological assessment, and cytokine concentrations in bronchoalveolar lavage (BAL) and plasma in 24 h from induction of acid aspiration acute lung injury in wild-type mice

Treatment	Wild-type mice with acid aspiration acute lung injury					
	24 h					
	Micro-CT scan	Histology		Cytokines		
	Change in the extent of alveolar collapse between 1 and 24 h (%) (*n*)	Alveolar sero-fibrinous exudate (visual score) (*n*)	Alveolar hemorrhage (visual score) (*n*)	BAL CXCL1 (pg/ml) (*n*)	BAL TNF-α (pg/ml) (*n*)	Plasma PTX3 (ng/ml) (*n*)
PBS	0.0 ± 10.9 (21)	1.0 [0.1–3.2] (7)	0.3 [0.0–3.2] (7)	35.8 [2.0–63.6] (18)	32.5 [32.0–73.0] (18)	296 [218–408] (15)
WT-MSCs	−8.1 ± 10.3* (17)	1.5 [0.2–1.9] (7)	0.1 [0.0–0.7] (7)	1.0 [0.1–30.1]^§^ (17)	29.0 [26.5–33.3]^§^ (17)	200 [155–275] (10)
PTX3^−/−^-MSCs	3.3 ± 10.3* (13)	0.1 [0.0–0.3] (5)	0.3 [0.0–0.9] (5)	34.5 [28.0–40.8] (9)	21.0 [15.8–25.8]^§^ (9)	275 [244–372] (15)
*p* value ANOVA or Kruskal–Wallis	0.01	0.07	0.50	<0.05	<0.05	0.142

*CXCL1* keratinocyte chemoattractant, *TNF-α* tumor necrosis factor-α, *PTX3* pentraxin 3

*Dunnett’s post hoc *p* < 0.05 vs. PBS

^§^Dunn’s post hoc *p* < 0.05 vs. PBS

**Fig. 4:**
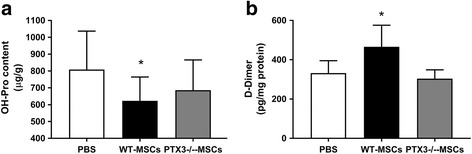
Effects of WT-MSCs on the fibrotic evolution of acid aspiration acute lung injury. Collagen deposition (OH-proline assay) (**a**) in lung tissue in the experimental groups in 2 weeks: WT-MSCs decreased presence of fibrotic tissue while PTX3^−/−^-MSCs could not (ANOVA *p* < 0.05 [A]; *Dunnett’s post hoc *p* < 0.05 vs. PBS. PBS *n* = 16; WT-MSCs *n* = 12; PTX3^−/−^-MSCs *n* = 10)). Decreased fibrotic evolution by WT-MSCs was likely mediated by improved fibrinolysis (**b**) in the lungs over the days following acid aspiration acute lung injury (ANOVA *p* < 0.01 [B]; *Dunnett’s post hoc *p* < 0.05 vs. PBS. PBS *n* = 5; WT-MSCs *n* = 5; PTX3^−/−^-MSCs *n* = 6) (PBS = acid aspiration acute lung injury + intraperitoneal (i.p.) PBS treatment in 1 h; WT-MSCs = acid aspiration acute lung injury + i.p. wild-type MSCs treatment in 1 h; PTX3^−/−^-MSCs = acid aspiration acute lung injury + i.p. PTX3-deficient MSCs treatment in 1 h)

In this study, we showed that treatment by i.p. WT-MSCs administered 1 h after acid aspiration attenuated the evolution of fibrosis, as demonstrated by lower collagen deposition (OH-Pro assay) in 2 weeks (Fig. 4a) in comparison to mice treated by PBS. In 1 week, d-dimer concentration was significantly increased in the lungs of mice treated with WT-MSCs (*p* < 0.001, Fig. 4b) in comparison to PBS, suggesting that dampening of long-term fibrotic evolution might have followed both reduced inflammation and enhanced fibrinolysis by WT-MSCs in the days after injury.

### Systemic deployment of WT-MSCs in 24 h

In an effort to evaluate whether i.p. WT-MSCs migrate systemically in mice with acid aspiration acute lung injury, we performed western blot analysis to detect GFP^+^ WT-MSCs presence in the lungs, spleen, liver, and peritoneal lavage in 24 h. Additional file 1: Figure S3 shows actual blots with no apparent signal of GFP^+^ WT-MSCs presence in the lungs, spleen, and liver as opposed to positive controls. In the peritoneal lavage, instead, WT-MSCs were still present in 24 h but by lower intensity, probably because, as previously shown [23], they formed aggregates and adhered to the peritoneal cavity walls.

### Lack of PTX3 in MSCs reduces early and long-term protection from acid aspiration acute lung injury

PTX3^−/−^-MSCs were isolated from the bone marrow of PTX3-knockout mice. The extensive characterization of PTX3^−/−^-MSCs demonstrated that these cells did not seem to modify their in vitro phenotypical and functional properties [18] (Additional file 1: Figure S1). In 24 h, treatment by PTX3^−/−^-MSCs ameliorated oxygenation only to a lesser extent than WT-MSCs (Fig. 2). Reduced short-term effects on oxygenation in comparison to WT-MSCs were paralleled by less effective reduction of wet-to-dry lung weight ratio by PTX3^−/−^-MSCs (Fig. 2a, b, e) and the absence of effects of PTX3-deficient cells on radiological signs of regional lung collapse and edema (Table 1). Histology found reduction of lung injury, albeit non-significant (Table 1). In summary, PTX3^−/−^-MSCs seemed less effective than WT-MSCs in limiting formation of lung edema in 24 h after acid aspiration. At variance from WT-MSCs, treatment with PTX3^−/−^-MSCs did not reduce total cell and neutrophil count (Fig. 2) as well as CXCL1 levels in the alveolar space (Table 1). Thus, the more limited effectiveness of PTX3^−/−^-MSCs in enhancing lung recovery after acid aspiration acute lung injury might have been related to ineffective reduction of the acute inflammatory processes. Moreover, PTX3^−/−^-MSCs could not modulate fibrinolysis in the days following injury nor impact the long-term fibrotic evolution, as demonstrated by unchanged levels of d-dimer and OH-proline in comparison to PBS (Fig. 4a, b). However, WT- and PTX3^−/−^-MSCs did not seem to modulate activity of MMP13 in 1 week (Additional file 1: Figure S2) to impact remodeling and fibrosis.

### Effects of study treatments on PTX3 knockout mice with acid aspiration acute lung injury

Extent of lung injury was similar between WT and PTX3^−/−^ mice (*t* test in 24 h in WT-mice + PBS vs. PTX3^−/−^-mice + PBS: PaO_2_, *p* = 0.151; wet-to-dry lung weight, *p* = 0.099). When administered to PTX3^−/−^-mice: WT-MSCs improved lung function and reduced fibrosis, but the difference with PBS was non-significant (Additional file 1: Table S3); PTX3^−/−^-MSCs induced a further non-significant reduction of the alveolar–arterial gradient and of wet-to-dry lung weight ratio, while PaO_2_ and fibrosis worsened in comparison to WT-MSCs (Additional file 1: Table S3). Thus, endogenous PTX3 might collaborate in the protective effects of WT-MSCs from fibrosis, while it might limit their effectiveness in reducing lung edema.

More results are provided in the Additional files of this article.

## Discussion

Study’s main findings can be summarized as follows: WT-MSCs dampen short- and long-term sequelae of acid aspiration acute lung injury in mice in terms of improved oxygenation, reduced edema causing lung collapse, and reduced fibrotic evolution, likely by fine-tuning the acute inflammatory reaction and the subsequent fibrinolysis and tissue repair process; moreover, lack of PTX3 gene in MSCs and in the injured host might reduce the beneficial effects of MSCs.

In the present study, we administered i.p. WT-MSCs 1 h after intratracheal instillation of hydrochloric acid, potentially reproducing real-life treatment of ARDS caused by aspiration of gastric contents [1, 12], one of the major direct causes of ARDS [24, 25] with a mortality rate around 35–40% and significant long-term fibrosis [1]. In 24 h from injury, we could show multiple short-term beneficial effects of WT-MSCs: as previously shown [7, 26], MSCs seemed to reduce the early inflammatory reaction in the lungs and to avoid excessive response and additional damage. In our study, indeed, MSCs dampened leukocyte trafficking through the alveolar–epithelial barrier as well as their activation and release of primary inflammatory cytokines. In turn, as testified by oxygenation, wet-to-dry and CT scan data, this led to decreased accumulation and/or improved clearance of lung edema and inflammatory cells in the alveolar and third-space compartments and to attenuated extent of alveolar collapse. However, histology did not improve after WT-MSCs administration, maybe due to insufficient numerosity; similarly, protein content in BAL was not reduced by MSCs, but this could have followed direct extravasation after acid-induced physical disruption of the alveolar–epithelial integrity. In our model, both lungs showed physiologic alterations, thus indicating that the left lung could completely compensate for the ventilation needs of the animals (Additional file 1: Table S2) [27].

In 2 weeks from acute lung injury onset, we also showed decreased long-term collagen deposition in the lungs associated with treatment by WT-MSCs. Moreover, the long-term reduction of fibrosis was preceded by increased fibrinolysis in 1 week. Our data, in keeping with recent literature [7, 9, 20], seem to suggest that the beneficial effects of MSCs on the fibrotic evolution of acute lung injury might include reduction of the acute-phase inflammatory reaction and reduced fibrosis in 2 weeks. Moreover, decreased respiratory effort during the early phases induced by improved gas exchange could have reduced interstitial lung edema [28] and the risk of additional ventilation-induced lung injury (VILI) and fibrosis [13]. In summary, it would be tempting to say that ours and the previous data indicate that MSCs might be regarded as personalized modular therapies limiting short- and long-term acute lung injury severity by fine-tuning inflammation and tissue remodeling. However, to date, whether these hypotheses hold true and will translate in improved mortality and long-term quality of life in human ARDS remains to be determined.

PTX3 is a marker of severity in human ARDS [29], and experimental models showed that PTX3 is as key determinant of the evolution, morbidity, and mortality of acute lung injury [30, 31]. A recent study by Cappuzzello and colleagues showed that while WT-MSCs improved tissue repair in experimental wound healing, PTX3^−/−^-MSCs could not [18]. Similarly, we showed that the early dampening of leukocyte migration and release of pro-inflammatory cytokines in the injured lungs by WT-MSCs could not be replicated when PTX3^−/−^-MSCs were adopted. This likely led to poorer effects on oxygenation and wet/dry ratios and no improvement in the CT scan analysis of lung collapse as well as no decrease in inflammatory cells and acute-phase primary cytokines in the BAL. The positive effect of PTX3^−/−^-MSCs treatment on the TNF-α levels may depend on the anti-inflammatory role of TSG6 [32, 33], which is highly expressed in PTX3^−/−^-MSC (Additional file 1: Figure S1). Previous studies in a mice model of bilateral acid aspiration lung injury showed that interaction between PTX3 and P-selectin is crucial for regulation of leukocyte recruitment with consequences on cytokine production and lung injury [23], and similar mechanisms might underlie lack of lung protection by PTX3^−/−^-MSCs. Moreover, we described that long-term fibrinolysis and subsequent fibrotic evolution could not be prevented by PTX3^−/−^-MSCs, possibly suggesting PTX3-mediated enhancement of lung tissue repair by WT-MSCs [18, 20]. On the other hand, our data indicate that the beneficial effects exerted by WT-MSCs are associated with a reduction in plasma PTX3, as if improvement of lung injury preceded modification of endogenous PTX3 production. However, lack of endogenous PTX3 seemed to reduce WT-MSCs effects (Additional file 1: Table S3), maybe by impairment of local cell-to-cell interaction. Our results do not generate a clear hypothesis on the role of PTX3 as molecular determinant of the lung protection exerted by MSCs, and further studies are warranted, maybe exploring other etiologies and time-points.

In our study, we could not detect presence of WT-MSCs in the liver, spleen, or lungs in 24 h, while in keeping with previous findings [34], a signal was still present in peritoneal lavage (Additional file 1: Figure S3). On the other hand, since levels of circulating PTX3 were lower and lung fibrinolysis was increased after administration of WT-MSCs, we might speculate possible migration and direct effect of MSCs at the site of injury but this cannot be concluded with any confidence. In summary, our data are not definitive to elucidate whether i.p. WT-MSCs act through paracrine vs. direct mechanisms.

This study suffers by a number of relevant limitations: as most of the measures required sacrifice of the animals (e.g., BAL), we could not assess in the same animal all the effects at different time-points but each effect was assessed in a subset of animals receiving the same injury and therapy, which might have introduced some heterogeneity. We only examined three time-points (i.e., 24 h and 1 and 2 weeks), which might have prevented us from description of other effects of WT-MSCs or PTX3^−/−^-MSCs in acid aspiration acute lung injury. Apart from resources limitation, our choice was based on previous observations on the time-course of the studied animal model [12]. We described reduced effectiveness of WT-MSCs induced by lack of PTX3 only in a murine model of non-infective acid aspiration lung injury and translation of these findings to other etiologies (e.g., infective pulmonary ARDS) and/or to the clinical setting warrants extreme caution. While we could determine significant effects of PTX3 presence in MSCs in 1 week to modulate fibrosis, the downstream effects of PTX3 presence in MSCs during the early acute phase (e.g., modulation of leukocyte recruitment by binding with P-selectin) remains to be elucidated. We did not evaluate the alteration of the alveolar–capillary permeability from a more molecular point of view (such as expression of tight or adherent junction proteins), but only by measuring the lung edema following such alterations (wet-to-dry ratio and the CT scan analysis). Besides the standard histological analysis, we did not perform more quantitative approach using stereological assessment of the tissue injury, and this might have limited our possibilities to describe more significant differences. This is a preliminary study: the small number of mice used and lack of other administration routes could have reduced significant differences. The experiments on PTX3^−/−^ mice were subsequent and separate from those on WT mice. Finally, volume of fluid instilled i.p. (i.e., 200 μl) might have induced cardiovascular impairment favoring pulmonary edema.

## Conclusions

The results presented here suggest that i.p. adoptive transfer of MSCs enhances short- and long-term lung recovery when cells are administered 1 h after onset of acute lung injury. PTX3, an acute-phase inflammatory mediator, might play a role in the lung protection exerted by MSCs, in particular against fibrosis, but this needs further clarification. Studies on the molecular mechanisms of actions of MSCs as well as on the risks associated with their administration should still proceed in parallel with ongoing translational studies [35]. In particular, PTX3 genetic polymorphisms have been associated with risk of microbial infections [36–38], and it will be important to assess whether PTX3 polymorphisms are associated with outcome in ARDS and in clinical trials aimed to assess the potential of MSCs.

## Additional file

Additional file 1: Detailed methods and additional results. (DOCX 838 kb)

## Abbreviations

ANOVA: One-way analysis of variance
ARDS: Acute respiratory distress syndrome
BAL: Bronchoalveolar lavage
CXCL1: Keratinocyte chemoattractant
GFP^+^: Green fluorescent protein-positive
i.p.: Intraperitoneal
MMP13: Matrix metalloproteinase 13
MSCs: Mesenchymal stem cells
OH-Pro: Hydroxyproline
PBS: Phosphate-buffered saline
PTX3: Pentraxin 3
PTX3^−/−^-MSCs: PTX3-deficient MSCs
TNF-α: Tumor necrosis factor-α
TSG-6: Tumor necrosis factor-stimulated gene 6
WT-MSCs: Wild-type MSCs
