# Supplementary material for: Intraperitoneal adoptive transfer of mesenchymal stem cells enhances recovery from acid aspiration acute lung injury in mice
Source: Intensive Care Med Exp. 2017 Mar 6;5:13. doi: 10.1186/s40635-017-0126-5 (PMC5339261; doi:10.1186/s40635-017-0126-5)
Supplement: Additional file 1: — Detailed methods and additional results. (DOCX 838 kb) [file 40635_2017_126_MOESM1_ESM.docx]

**Intraperitoneal adoptive transfer of mesenchymal stem cells enhances recovery from acid aspiration acute lung injury in mice**

Tommaso Mauri, Vanessa Zambelli, Claudia Cappuzzello, Giacomo Bellani, Erica Dander, Marina Sironi, Vittoria Castiglioni, Andrea Doni, Alberto Mantovani, Cecilia Garlanda, Giovanna D’amico, Antonio Pesenti.

**Additional files**

**DETAILED METHODS**

**Animals.** Male C57BL/6J mice (22-25 g; Harlan Laboratories, Udine, Italy) and PTX3^-/-^-mice generated on a C57BL/6J or 129/Sv inbred genetic background matched with the appropriate wild-type controls as previously described [E1] were maintained under standard laboratory condition at University of Milan-Bicocca, Monza, Italy. Procedures involving animals and their care were conducted in conformity with the institutional guidelines complying with national and international laws and policies. The experimental protocol was submitted to the Italian Ministry of Health and approved by the Animal Care Unit of the University of Milan-Bicocca, Monza, Italy.

**Isolation of MSCs.** Murine MSCs were isolated from 8 week-old female C57Bl/6 wild type (WT) and from PTX3^-/-^-mice by the following procedures [E1]: bone marrow cells were collected by flushing the femurs and tibias with the medium and cultivated in 25 cm^2^ tissue culture flasks at a concentration of 2 x 10^6^ cells/cm^2^ using complete Dulbecco modified eagle medium (Lonza, Braine-L’Alleud, Belgium) supplemented with 20% heat inactivated fetal bovine serum (Biosera, Ringmer, United Kingdom), 2mM glutamine (Lonza, Braine-L’Alleud, Belgium), 100 U/ml penicillin/streptomycin (Lonza, Braine-L’Alleud, Belgium) and 100 μg/ml gentamicin (Euroclone, Pero, Italy). Then, cultures were incubated at 37°C in a 5%-CO_2_ atmosphere and, after 48 hours, the non-adherent cells were removed. When the cells reached 70–80% confluence, the adherent cells were trypsinized (0.05% trypsin at 37°C for 3 min), harvested and expanded in larger flasks at a concentration of 1.5-2.0 x 10^4^ cells/cm^2^ until passage 3, then maintained at 3000 cells/cm^2^. At passage 5, MSCs were screened by flow cytometry for the expression of CD106, CD45, CD117, CD73, CD105, MHC-I, SCA-1 and CD11b (BD Pharmingen, Oxford, UK) and for their capacity to differentiate into adipocytes and osteoblasts. Fresh MSCs at passages 5 to 7 were used for the present study. Previous studies showed that PTX3^-/-^-MSCs were similar to WT-MSCs in their ability to grow spontaneously, undergo mesengenic differentiation and express MSC cell-surface antigens (E2). Moreover, PTX3^-/-^-MSCs do not store or release PTX3 [E2] while, at variance, they produce normal levels of TSG-6 (i.e., one of the key mediators of MSCs) (see below and **Additional file 1**: Figure S1) [E3].

**Experimental protocol.** Acid aspiration acute lung injury was induced as already described [E4]. Briefly, mice were anesthetized with Tribromoethanol (400 mg/kg) (Sigma-Aldrich, Milan, Italy) by intra-peritoneal injection and connected to a rodent ventilator (Inspira ASV, Harvard Apparatus, Holliston, MA). Mechanical ventilation was set as follow: tidal volume of 15 ml/kg, respiratory rate of 130 breaths/min, positive end expiratory pressure (PEEP) of 2 cmH_2_O, fraction of inspired oxygen (FiO_2_) of 1, inspiratory on expiratory (I:E) ratio of 35%. Immediately after intubation, 1.5 ml/kg of 0.1M hydrochloric acid (HCl) was instilled into the right lung and, after 10 minutes, animals were extubated and placed in an oxygenated chamber. One hour later, mice received IP injection of 200 µl of sterile Phosphate Buffered Saline (PBS), or 1x10^6^ WT-MSCs or 1x10^6^ PTX3^-/-^-MSCs (both in equal volume of 200 µl). Then, all the following physiological measures were performed at 24 hours and/or 2 weeks from induction of acid aspiration acute lung injury.

The murine anatomy facilitate the cannulation of the right bronchus, however at the moment of the sacrifice we evaluated macroscopically which lung was affected, in order to ensure that acid instillation was only in the right lung and we didn’t exclude anyone, as no mice had the left lung affected.

**Arterial blood gas measurement.** A blood sample was withdrawn at 24 hours and 2 weeks from the left ventricle and analyzed (0.1 mL) with an I-STAT 1 portable analyzer (Oxford Instruments S.M., Burke e Burke, Menfis Biomedica, Milan, Italy).

**Wet to dry ratio.** At 24 hours and 2 weeks, lungs were excised, rinsed, blotted and immediately weighted (wet weight). Then lungs were kept at 70°C in a ventilated oven and, after 72 hours, weighted again (dry weight).

**Micro-Computed Tomography imaging.** Right after acid instillation and at 24 hours, mice were anesthetized, oro-tracheally intubated, mechanically ventilated with the above-mentioned settings and subjected to a micro-CT scan (SkyScan 1176, Bruker, Belgium) with the following parameters: voltage 58 kV, current 430 µA and resolution 35 µm. The scans were acquired over 4 minutes. Images were reconstructed and analyzed with the dedicated scanner software (NRecon v.1.6.9 and CTAn v.1.13, SkyScan, Bruker, Belgium) to obtain the percentage of non-aerated lung tissue at each time-point. Briefly, lungs mask was manually identified and areas of increased density indicating lung edema were measured as percentage of the whole lung volume. Then, the difference of the percentage of abnormal dense areas at 24 hours from acid instillation and those at the moment of the lung injury induction (T0) was calculated. Investigators who performed the analysis were blinded to the experimental groups.

**Histology.** At 24 hours and 2 weeks, the lungs were excised, fixed in 4% formaldehyde for 24 h (at a pressure of 20 cmH_2_O for the first 30 min) and then paraffin embedded and sectioned. Transverse sections of 5 µm were obtained by cutting the lungs from apex to base and then stained with hematoxylin and eosin (Sigma-Aldrich, St. Louis, MO). Histopathology examination was performed according to previous study [E4], evaluating alveolar serofibrinous exudate with hyaline membranes formation and alveolar hemorrhage. Severity and extension of each pathologic finding were scored by an experienced pathologist (VC) blinded to treatment received by each animal for severity as 0 (absent), 1 (mild), 2 (moderate), 3 (marked) and for extension as 0 (absent), 1 (>0% and ≤25%), 2 (>25% and ≤50%), 3 (>50%). An average score per finding per each lung was derived and expressed as the product of extent and severity.

**Bronchoalveolar lavage (BAL).** At 24 hours and 2 weeks, after animal exsanguination, the thorax was opened and macroscopic observation of the lungs allowed identification of the side where acid injury was induced, as hemorrhagic and non-recruitable area. Then, BAL was performed separately for each lung, by clamping alternatively the left and right bronchus. Lavage was performed three times for each lung, with 600 or 400 µl of lavage solution (0.9% saline solution and protease inhibitor) respectively for the right and left one. The BAL samples obtained were then mixed and centrifuged for 10 minutes, 1500 rpm, 4°C; the supernatant was then stocked at -80°C for subsequent dosages. The cell pellet was resuspended in 500 µl PBS. 100 µl were put in 200 µl of Turk for total leukocyte count in a Burker chamber, while 100 µl were centrifuged by a Cytospin (Centrifuge, MPW-351R, MPW, Warsaw, Poland) and then colored with a Diff-Quick kit (Medion Diagnostics, Düdingen, Switzerland), that stains differently nucleus and cytoplasm, thus allowing to obtain the differential cell count.

**Protein concentration.** Protein concentration in BAL fluid was measured by BCA (bicinchoninic acid) method at 24 hours and 2 weeks. Briefly, 200 µl of reagent (composed by bicinchoninic acid and CuSO_4_, 1:50) was added to samples. Standard curve was constructed with increasing concentrations of bovine serum albumin. Then a spectrophotometer reading was performed at 570 nm.

**Cytokines measure.** At 24 hours, keratinocyte chemoattractant (CXCL1, previously named KC) and tumor necrosis Factor-α (TNF-α) levels were assayed in BAL by ELISA according to the manufacturer’s instructions (R&D Systems, Minneapolis, MN), while PTX3 levels were measured in plasma by ELISA as previously described [E5].

**D-dimer assay.** D-dimer levels were measured in lung lysate at one week by ELISA, following manufacturer’s instructions (AMS Biotechnology, Bioggio-Lugano, Switzerland) as reported [E6].

**Markers of downstream WT-MSCs effects.** At 1 week, matrix metalloproteinase 13 (MMP13, an enzyme that participates in collagen degradation) [E7] was assayed by western blot in lung lysate. Briefly, 30µg of lung protein extract were loaded on 8-15% SDS-PAGE (Bio-Rad Laboratories, Segrate [MI], Italy) and a goat polyclonal anti-MMP13 (0.4µg/ml; R&D Systems, Minneapolis, MN) was used. Recombinant MMP-13 (50ng/lane; R&D Systems, Minneapolis, MN) and a lysate obtained from murine fibrosarcoma cells (5.10^5^/lane) were used as positive control.

**Hydroxyproline (OH-Pro) assay.** At 2 weeks, lungs were excised and stored at -80°C. Then, collagen content was measured with the OH-Pro assay [E8]. We used the conventional method, which entails lung tissue homogenization and hydrolysis with 6N HCl at 120°C, followed by chloramine T and Ehrlich’s solution addition to samples for the OH-proline oxidation and a colorimetric reaction. Finally, absorbance was measured at 550nm.

**Analysis of TSG-6 expression in WT - and PTX3^-/-^-MSCs by Real Time PCR.** After culture, MSCs were digested with Trizol Reagent (Invitrogen, Foster City, CA, USA). RNA was extracted and was reverse transcribed, using a cDNA reverse transcription kit (Invitrogen, Foster City, CA, USA). The presence of TSG-6 (forward 5’-aggcagccagaaaaattgg; reverse 5’-cacaatggggtatccgactc) was determined by qRT-PCR using UPL-Light Cycler Technology (Roche Diagnostics, Milan, Italy). The Ct values for housekeeping gene HPRT (Hypoxanthine-guanine phosphoribosyltransferase) were used to normalize the expression level of the gene of interest using the ΔΔCt method.

**Western blotting to detect systemic deployment of MSCs.** Slightly smaller groups of WT-mice with the same acid aspiration acute lung injury were used to search MSCs’ presence in the lungs, liver, spleen and peritoneal cavity at 24 hours by western blotting of green fluorescent protein-positive (GFP^+^) WT-MSCs. In particular, mice underwent induction of acid aspiration acute lung injury as described above, and 1 hour later they were treated by i.p. 1x10^6^ GFP^+^ WT-MSCs or 200 µl of sterile PBS or intra-tracheal 1x10^6^ GFP^+^ WT-MSCs. The mouse receiving intra-tracheal GFP^+^ WT-MSCs died right after, while, at 24 hours, mice which received i.p. GFP^+^ WT-MSCs and PBS were euthanized via exsanguinations and whole lungs, spleen, liver and peritoneal lavage were collected and homogenized in 50mM Tris-HCl pH 7.5 containing 2mM EGTA, 1mM PMSF, 100KU Aprotinin, 1% Triton-X100 (all from Sigma-Aldrich, St. Louis, MO) and complete protease inhibitor cocktail (Roche Diagnostics, Milan, Italy). DC TM Protein Assay measured total proteins, according to manufacturer’s instructions (Bio-Rad Laboratories S.r.l., Segrate [MI], Italy). Western blot analysis for GFP (rabbit polyclonal anti-GFP, 1:1500; Abcam, Cambridge, UK) was performed after loading 5 μg of lung protein extracts on 4-20% SDS-PAGE Precast Mini gels (Bio-Rad Laboratories S.r.l., Segrate [MI], Italy). Optical density values were internally normalized by mouse monoclonal anti-β-actin (1:20000; Sigma-Aldrich, St. Louis, MO) and Red Ponceau (Sigma-Aldrich, St. Louis, MO) and further corrected for the value of control considered equal to 1.

**Statistical analyses.** Data are expressed as mean ± standard deviation (SD) if normally distributed and as median [interquartile range] when non-normally distributed (Shapiro-Wilk test). Differences in variances between treatment groups were assessed by one-way analysis of variance (ANOVA) and Dunnett post hoc test versus PBS group or by Kruskal–Wallis test and Dunn post hoc test versus PBS group, as appropriate. Differences in physiologic variables measured in right vs. left lung were assessed by t-test or Mann-Whitney U test, as appropriate. P values of less than 0.05 were considered as statistically significant. Statistical analysis was performed by IBM SPSS Statistics Version 19 (IBM Corp., Armonk, NY) by Sigmaplot 11.0 (Systat software Inc., Chicago, IL) and by Prism 7 for Windows (GraphPad Software Inc., La Jolla, CA).

**ADDITIONAL RESULTS**

**Survival.** The survival rate in this model was quite high in all the experiments: at 24 hours from acid instillation, 95% of mice treated by PBS, 98% by WT-MSCs and 100% by PTX3^-/-^-MSCs survived. At 2 weeks, 87% of mice in PBS group, 96% in WT-MSCs group and 87% in PTX3^-/-^-MSCs group survived.

**Effects of study treatments on right vs. left lungs.** Differential analysis of the effects of study treatments on the 2 lungs showed that, as expected, the right side (where acid aspiration was induced) was sicker than the left lung in terms of edema, inflammatory cells recruitment and long-term fibrosis (**Additional file 1**: Table S2). Nonetheless, the left lung wasn’t spared from injury and the beneficial effects of WT-MSCs were evident on both sides (**Additional file 1:** Table S2).

**TABLES**

**Additional file 1: Table S1. Arterial partial CO_2_ tension and pH in mice with acid aspiration acute lung injury at 24 hours.**

| Treatment | Wild type mice with acid aspiration acute lung injury | |
| --- | --- | --- |
|  | **24 hours** | |
|  | **PaCO_2_**  **(mmHg)** | **pH** |
| PBS | 47.1 [39.6-48.1] | 7.158 [7.065-7.160] |
| WT-MSCs | 46.2 [43.3-49.8] | 7.065 [7.003-7.097] |
| PTX3^-/-^-MSCs | 50.6 [46.1-53.2] | 7.134 [7.109-7.172] |
| P-value  Kruskal Wallis | 0.21 | 0.05 |

**Additional file 1: Table S2. Measures of lung edema, broncho-alveolar lavage (BAL) cells and OH-Proline content in the right vs. left lung in wild-type mice with acid aspiration acute lung injury.**

| Treatment | Wild type mice with acid aspiration acute lung injury | | | | | | | |
| --- | --- | --- | --- | --- | --- | --- | --- | --- |
|  | **24 hours** | | | | | | **2 weeks** | |
|  | **Wet to dry lung weight ratio** | | **Total BAL cells count**  **(cells/µl)** | | **Total BAL PMNs count**  **(cells/µl)** | | **OH-Pro content**  **(µg/g lung)** | |
|  | **Right lung** | **Left lung** | **Right** | **Left** | **Right** | **Left** | **Right** | **Left** |
| PBS | 6.00* ± 0.55 | 4.81 ± 0.44 | 270^#^  [228-328] | 148  [133-260] | 158^#^  [122-213] | 31  [22-146] | 857* ± 319 | 656 ± 236 |
| WT-MSCs | 5.60* ± 0.50 | 4.56 ± 0.25 | 149  [106-190] | 104  [84-139] | 68^#^  [53-109] | 37  [24-68] | 613 ± 196 | 649 ± 262 |
| PTX3^-/-^-MSCs | 5.72* ± 0.65 | 4.64 ± 0.35 | 220  [166-269] | 202  [138-297] | 108  [72-174] | 57  [22-186] | 698 ± 274 | 645 ± 356 |

* T-test: p<0.05 vs. Left

^#^ Mann-Whitney U test: p<0.05 vs. Left

**Additional file 1: Table S3. Oxygenation, lung edema and collagen deposition in PTX3^-/-^ mice with acid aspiration acute lung injury treated by PBS and WT- or PTX3^-/-^-MSCs.**

| Treatment | PTX3^-/-^-mice with acid aspiration acute lung injury | | | |
| --- | --- | --- | --- | --- |
|  | **24 hours** | | | **2 weeks** |
|  | **PaO_2_**  **(mmHg)** | **A-a Gradient**  **(mmHg)** | **Wet/Dry lung weight ratio** | **OH-Pro content**  **(µg/g lung)** |
| PBS | 87 [67-108] | 20 [0-32] | 5.40 ± 0.63 | 1001 ± 458 |
| WT-MSCs | 97 [89-109] | 7 [0-24] | 5.29 ± 0.40 | 940 ± 505 |
| PTX3^-/-^-MSCs | 78 [71-99] | 1 [0-40] | 4.76 ± 0.30 | 1156 ± 312 |
| P-value  ANOVA or Kruskal Wallis | 0.309 | 0.837 | 0.350 | 0.604 |

**FIGURES**

**Additional file 1: Figure S1. Production of TSG-6 in wild type (WT) and Pentraxin 3 knockout (PTX3^-/-^) mesenchymal stem cells (MSCs).** Previous studies showed that PTX3^-/-^-MSCs were similar to WT-MSCs in their ability to grow spontaneously, undergo mesengenic differentiation and express common MSCs’ markers. Moreover, this plot shows that PTX3^-/-^-MSCs did not store or release PTX3 while, at variance they tended to produce higher levels of TSG-6 (i.e., a key mediator of MSCs’ actions) compared to WT-MSCs.


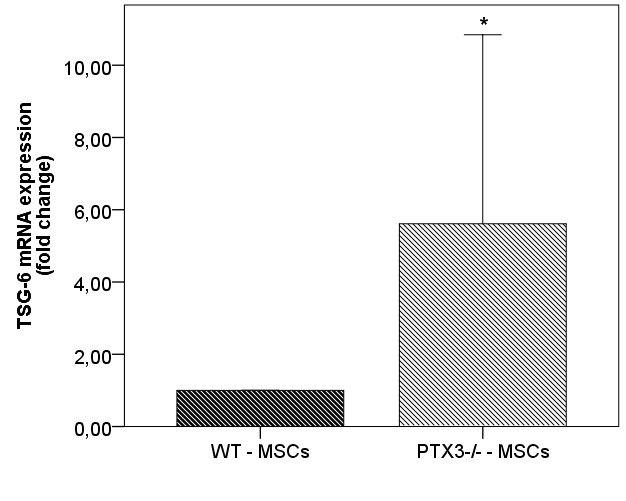


**Additional file 1: Figure S2. Western blots of Metalloproteinase 13 (MMP 13) at 1 week.** Wild type (WT-) and Pentraxin 3 knockout (PTX3^-/-^) mesenchymal stem cells (MSCs) didn’t seem to modulate activity of matrix metalloproteinase 13 (MMP13, an enzyme that participates in collagen degradation) in comparison to treatment with Phosphate Buffered Saline (PBS).

KD

Fibrosarcoma

lysate

PBS

MSC wt

MSC *Ptx3^-/-^*


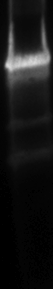

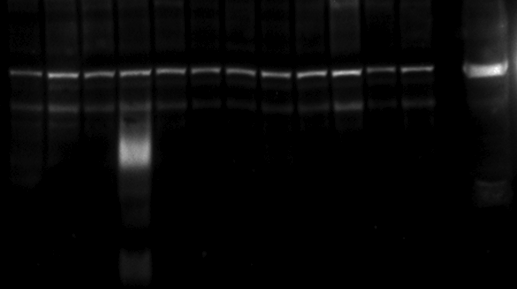


- MMP-13

Cleavage fragments

Purified MMP-13


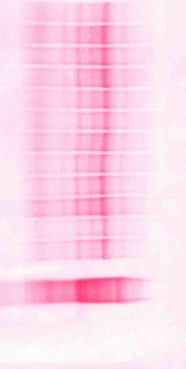


1 2 3 4

1 2 3 4

1 2 3 4

**Additional file 1: Figure S3. Systemic deployment of intra-peritoneal Mesenchymal Stem Cells.** At 24 hours, western blot analysis of lung tissue (A) shows intense signal from green fluorescent protein-positive (GFP+) mesenchymal stem cells injected intra-tracheally but not after intra-peritoneal administration. Similarly, at 24 hours, signal from intra-peritoneal GFP+ mesenchymal stem cells could not be detected (B) in the liver nor in the spleen, while (C) signal seems evident in peritoneal lavage (i.t. MSC = acid aspiration acute lung injury + intra-tracheal wild type GFP^+^-MSCs after 1 hour; PBS = acid aspiration acute lung injury + PBS after 1 hour; i.p. MSC = acid aspiration acute lung injury + intra-peritoneal wild type GFP^+^-MSCs after 1 hour; GFP^+^-MSC = positive control).

**
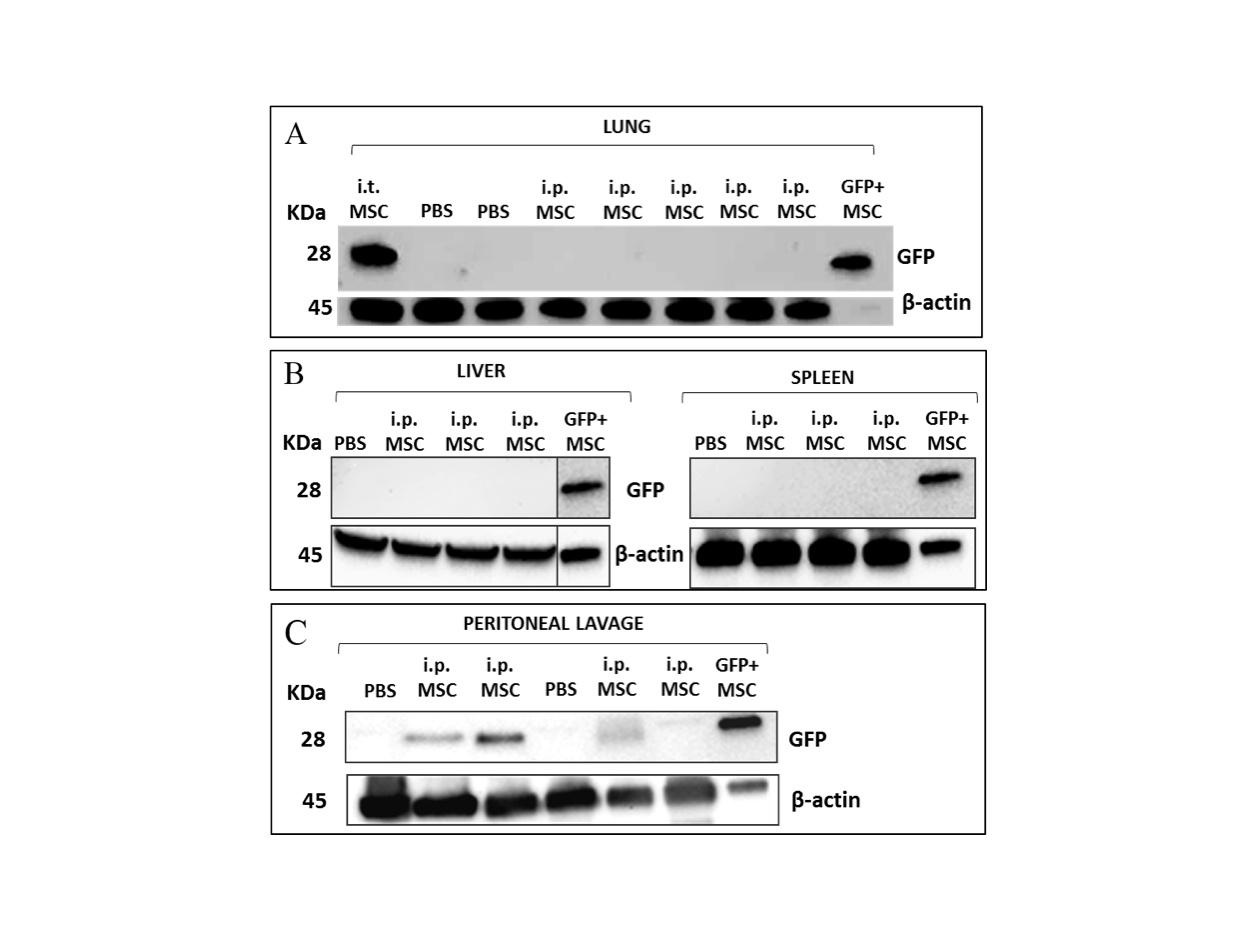
**

**Additional file 1: Figure S4. Histological methods.** Images of hematoxylin-eosin stained lung tissue sections of acid aspiration acute lung injury treated by PBS at 24 hours from injury showing presence of alveolar hemorrhage (top panel) and alveolar sero-fibrinous exudate (bottom) (200x, scale bar 100µm).

**
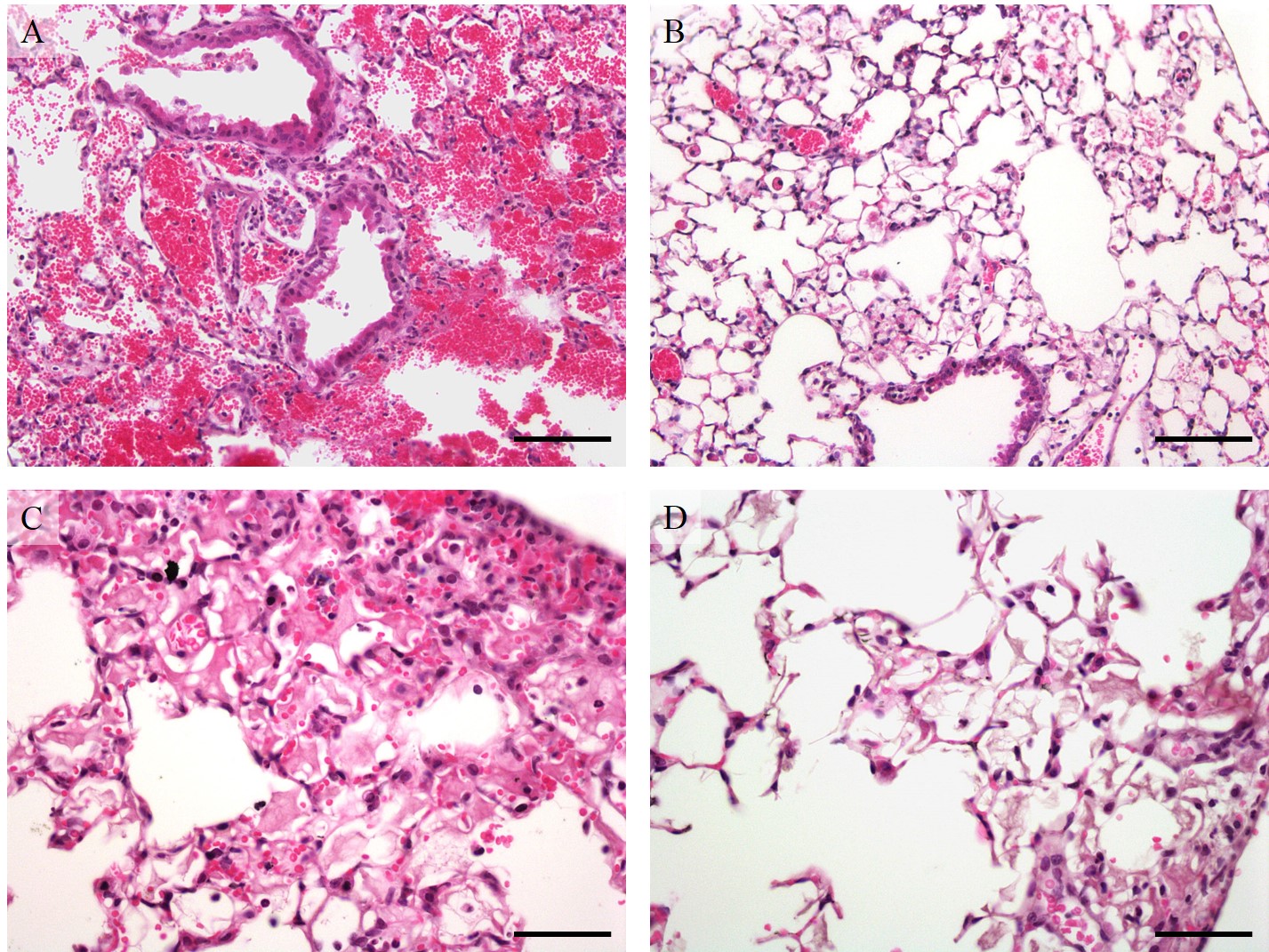
**

**REFERENCES**

E1. Deban L, Russo RC, Sironi M, Moalli F, Scanziani M, Zambelli V, Cuccovillo I, Bastone A, Gobbi M, Valentino S, Doni A, Garlanda C, Danese S, Salvatori G, Sassano M, Evangelista V, Rossi B, Zenaro E, Constantin G, Laudanna C, Bottazzi B, Mantovani A (2010) Regulation of leukocyte recruitment by the long pentraxin PTX3. Nat Immunol 11(4):328-334.

E2. Cappuzzello C, Doni A, Dander E, Pasqualini F, Nebuloni M, Bottazzi B, Mantovani A, Biondi A, Garlanda C, D'Amico G (2016) Mesenchymal stromal cell-derived PTX3 promotes wound healing via fibrin remodeling. J Invest Dermatol 136(1):293-300.

E3. Walter J, Ware LB, Matthay MA (2014) Mesenchymal stem cells: mechanisms of potential therapeutic benefit in ARDS and sepsis. Lancet Respir Med 2(12):1016-1026.

E4. Amigoni M, Bellani G, Scanziani M, Masson S, Bertoli E, Radaelli E, Patroniti N, Di Lelio A, Pesenti A, Latini R (2008) Lung injury and recovery in a murine model of unilateral acid aspiration: functional, biochemical, and morphologic characterization. Anesthesiology 108(6):1037-1046.

E5. Mauri T, Coppadoro A, Bombino M, Bellani G, Zambelli V, Fornari C, Berra L, Bittner EA, Schmidt U, Sironi M, Bottazzi B, Brambilla P, Mantovani A, Pesenti A (2014) Alveolar pentraxin 3 as an early marker of microbiologically confirmed pneumonia: a threshold-finding prospective observational study. Crit Care 18(5):562.

E6. Doni A, Musso T, Morone D, Bastone A, Zambelli V, Sironi M, Castagnoli C, Cambieri I, Stravalaci M, Pasqualini F, Laface I, Valentino S, Tartari S, Ponzetta A, Maina V, Barbieri SS, Tremoli E, Catapano AL, Norata GD, Bottazzi B, Garlanda C, Mantovani A (2015) An acidic microenvironment sets the humoral pattern recognition molecule PTX3 in a tissue repair mode. J Exp Med 212(6):905-925.

E7. Juncker-Jensen A, Lund LR (2011) Phenotypic overlap between MMP-13 and the plasminogen activation system during wound healing in mice. PLoS One 6(2):e16954.

E8. Tager AM, Kradin RL, LaCamera P, Bercury SD, Campanella GSV, Leary CP, Polosukhin V, Zhao LH, Sakamoto H, Blackwell TS, Luster AD (2004) Inhibition of pulmonary fibrosis by the chemokine IP-10/CXCL10. Am J Respir Cell Mol Biol 31:395-404.
